# Chemical Constituents and Evaluation of Antimicrobial and Cytotoxic Activities of *Kielmeyera coriacea* Mart. & Zucc. Essential Oils

**DOI:** 10.1155/2015/842047

**Published:** 2015-04-16

**Authors:** Carla de M. Martins, Evandro A. do Nascimento, Sérgio A. L. de Morais, Alberto de Oliveira, Roberto Chang, Luís C. S. Cunha, Mário M. Martins, Carlos Henrique G. Martins, Thaís da S. Moraes, Paulla V. Rodrigues, Cláudio V. da Silva, Francisco J. T. de Aquino

**Affiliations:** ^1^Nucleus of Research in Natural Products, Chemistry Institute, Federal University of Uberlândia, 38400-902 Uberlândia, MG, Brazil; ^2^Goian Federal Institute, Campus Morrinhos, 75650-000 Morrinhos, GO, Brazil; ^3^Nucleus of Research in Sciences and Technology, Laboratory of Research in Applied Microbiology, University of Franca, 14404-600 Franca, SP, Brazil; ^4^Institute of Biomedical Sciences, Laboratory of Trypanosomatids, Federal University of Uberlândia, 38400-902 Uberlândia, MG, Brazil

## Abstract

Many essential oils (EOs) of different plant species possess interesting antimicrobial effects on buccal microorganisms and cytotoxic properties. EOs of *Kielmeyera coriacea* Mart. & Zucc. were analyzed by gas chromatography coupled to mass spectrometry (GC-MS). The EO from leaves is rich in sesquiterpenes hydrocarbons and oxygenated sesquiterpenes. The three major compounds identified were germacrene-D (24.2%), (E)-caryophyllene (15.5%), and bicyclogermacrene (11.6%). The inner bark EO is composed mainly of sesquiterpenes hydrocarbons and the major components are alpha-copaene (14.9%) and alpha-(E)-bergamotene (13.0%). The outer bark EO is composed mainly of oxygenated sesquiterpenes and long-chain alkanes, and the major components are alpha-eudesmol (4.2%) and nonacosane (5.8%). The wood EO is mainly composed of long-chain alkanes and fatty acids, and the major components are nonacosane (9.7%) and palmitic acid (16.2%). The inner bark EO showed the strongest antimicrobial activity against the anaerobic bacteria *Prevotella nigrescens* (minimum inhibitory concentration-MIC of 50 *µ*g mL^−1^). The outer bark and wood EOs showed MICs of 100 *µ*g mL^−1^ for all aerobic microorganisms tested. The EOs presented low toxicity to Vero cells. These results suggest that *K. coriacea*, a Brazilian plant, provide initial evidence of a new and alternative source of substances with medicinal interest.

## 1. Introduction

The plants are part of our daily life and their essential oils (EOs) have been extracted from 3000 different sources, of which 200 to 300 have domestic, industrial, and medicinal use [1]. They have an important role in the protection of plants and pollination and their biological activities have been long related to antinociceptive, anticancer, antiphlogistic, antiviral, antioxidant, and antimicrobial activities [2–4].

The Brazilian cerrado (savannah) biome is a great source of medicinal plants, with several pharmacologically active species used in folk medicine, contributing to significant knowledge of bioactive compounds. The plants of the genus* Kielmeyera* (Clusiaceae) are endemic in South America and the* Kielmeyera coriacea* species is often documented primarily in savannahs. This species has economic interest for the production of wood, cellulose, and tannin for the leather industry [4]. From the bark of the plant, a tonic and emollient yellow resin can be extracted, which is used in folk medicine to treat toothache [5]. This species is rich in xanthones, substances that have pharmacological properties, such as antitumor, antifungal, antibacterial, and anti-inflammatory activities [6]. Several xanthones have been isolated from the stem bark of the plant and have showed great antifungal and antimicrobial activities [7–9].

The species* Kielmeyera coriacea* Mart. & Zucc is popularly known as* pau-santo* in Brazil. Organic extracts of different parts of this plant have been tested against oral microorganisms, and the inner bark extract showed promising results against aerobic and anaerobic oral microorganisms. However, chemical analysis or investigation of antimicrobial properties of the essential oil from leaves, barks, and wood of* K. coriacea* Mart. & Zucc have not been previously published. Our research group has reported that the* in vitro* assays with essential oils of some native plants from Brazil's Cerrado biome are promising antimicrobial agents against oral microorganisms. The essential oils studied exhibited a broad spectrum of action against different aerobic and anaerobic oral bacteria, or were selective to a particular group of oral bacteria with low toxicity. Additionally, the literature has also reported on the biological and pharmacological effects using different plant parts of the* K. coriacea* [10–19].

The aim of the present study is to evaluate the antimicrobial activity of essential oils from different parts of* K. coriacea* Mart. & Zucc. against Gram-positive and Gram-negative oral bacteria, traditionally involved with different oral illness, including dental caries and periodontal diseases [23–26].

## 2. Materials and Methods

### 2.1. Plant Material

The plant material was collected in Monte Alegre de Minas, Minas Gerais state, Brazil, (Latitude: 18°34′56.85′′S; Longitude: 49°2′52.61′′O) in July 2012 (dry season). A specialist identified the plant and a voucher specimen (57181) was deposited in the herbarium of the Federal University of Uberlândia, Brazil.

### 2.2. Essential Oil Extraction

The fresh material of* K. coriacea* (leaves, inner bark, outer bark, and wood) was cut into small pieces and about 400 g of each part was individually placed in a round-bottomed flask. The essential oil extraction was done by hydrodistillation using a Clevenger-type apparatus for 4 hours. Then, the oil obtained was extracted with dichloromethane (5 mL). The organic fraction was dried with anhydrous sodium sulphate, filtered, and kept in a closed vial under refrigeration (−10°C) for further analysis. The dichloromethane was removed for all biological assays with essential oils. The percentage yield was calculated relative to the dried mass of the initial sample.

### 2.3. Identification and Quantification of the EO

The compounds in the EO were identified by a GC-17A/QP-5000 gas chromatograph coupled to a mass spectrometer (Shimadzu, Kyoto, Japan) equipped with a 30-m DB-5 capillary column (5% phenyl, 95% polydimethylsiloxane, J&W, USA, 30 m × 0.25 mm × 0.25 *μ*m film thickness). The carrier gas used was Helium at a flow rate of 1.0 mL min^−1^; the detector and injector temperatures were 220°C and 240°C, respectively, and the injection volume was 1.0 *μ*L of a solution of 5 mg mL^−1^ of the EOs in CH_2_Cl_2_, using split mode (ratio 1 : 20). The oven temperature was programmed from 60°C to 240°C at a ramping rate of 3°C min^−1^. The electron impact energy was set at 70 eV, and fragments from 40 to 650 *m*/*z* were collected. The identification of the chemical constituents was carried out by comparison of the mass spectra obtained with those stored in libraries (Wiley7; Wiley229; Nist08; Nist08s; Nist27) and based on the calculated arithmetic indexes (AIc) and the arithmetic indexes (AI) reported in the literature [20–22]. The AIc were calculated using the equation AI(X) = 100PzC + 100[(*t*(X) − *t*(Pz))/(*t*(Pz + 1) − *t*(Pz))], which is based on retention times (*t*) of  linear alkane standards, which, by definition, have an AI equal to 100 × number of carbon atoms; X = compound at time *t*; PzC = number of carbon atoms of the alkane Pz, which runs just before X; Pz + 1 = alkane running just after X  [20]. For calculation of arithmetic indexes, a mix of linear alkanes C_8_–C_40_ and terpene standards (Sigma-Aldrich) was injected under the same conditions. Quantification was performed by gas chromatography equipment with detector flame ionisation (GC-FID, Shimadzu GC-2014), using the same capillary column DB-5 under the same conditions as for GC/MS, but nitrogen was used as the carrier gas. Quantification was obtained after normalisation of the peak areas from FID response. The experiments were done in triplicate.

### 2.4. Microbial Strains

The tested strains were obtained from the American Type Culture Collection (ATCC, Rockville MD, USA). The following microorganisms were used in the evaluation of the antibacterial activity of the essential oils:* Streptococcus mitis* (ATCC 49456),* Streptococcus mutans* (ATCC 25175),* Streptococcus sanguinis* (ATCC 10556),* Streptococcus sobrinus* (ATCC 33478),* Actinomyces naeslundii* (ATCC 19039),* Bacteroides fragilis* (ATCC 25285), and* Prevotella nigrescens* (ATCC 33563).

### 2.5. Antimicrobial Activity

The minimum inhibitory concentration (MIC) values of the essential oils were determined in triplicate by the microdilution broth method in 96-well microplates (TPP, EUA) [27]. The samples were dissolved in dimethyl sulfoxide (DMSO, Synth, São Paulo, Brazil; 8000 *μ*g mL^−1^), followed by dilution in tryptic soy broth (Difco, Detroit, MI, USA) for aerobic bacteria, and Schaedler broth (Difco), supplemented with hemin (5.0 *μ*g mL^−1^) and vitamin K1 (10.0 *μ*g mL^−1^) for anaerobic bacteria, in order to achieve concentrations ranging from 400 to 12.5 *μ*g mL^−1^. The final DMSO concentration was 4% (v v^−1^), and this solution was used as a negative control. The inoculum was adjusted for each organism to yield a cell concentration of 5 × 10^5^ colony forming units (CFU) per mL, according to the National Committee for Clinical Laboratory Standard (NCCLS) guidelines [28]. Chlorhexidine dihydrochloride (CHD, Sigma, Poole, Dorset, UK) was used as a positive control, and the concentrations ranged from 0.0115 *μ*g mL^−1^ to 5.9 *μ*g mL^−1^. The controls of sterility of TSB and Schaedler broths, chlorhexidine dihydrochloride sterility, sterility of the extracts, control culture (inoculum) and control DMSO were performed. The microplates (96 wells) with the aerobic microorganisms were closed with a sterile plate sealer and incubated aerobically at 37°C for 24 h. The anaerobic microorganisms were closed with a sterile plate sealer and incubated for 48–72 h in an anaerobic chamber (Don Whitley Scientific, Bradford, UK), in 5%–10% H_2_, 10% CO_2_, 80%–85% N_2_ atmosphere, at 37°C. After that, resazurin (Sigma, 30 *μ*L) in aqueous solution (0.01%) was added to indicate microorganism viability [27]. The MIC values were determined as the lowest concentration of the essential oils capable of inhibiting microorganism growth.

### 2.6. Cytotoxic Activity (CC_50_)

Samples of the essential oils were dissolved in methanol and diluted in DMEM (Dulbecco's modified Eagle's medium) culture medium supplemented to form a stock solution of 640 *μ*g mL^−1^. The cell viability test was performed with Vero cells (ATCC CCL 81) (kidney fibroblasts, African green monkey). For evaluation of cytotoxicity, the microplate dilution method was used. A solution containing 1 × 10^6^ cells in 10 mL supplemented DMEM was prepared, and 100 *μ*L of this solution was pipetted into each well, and then the plate was incubated for 6 h at 37°C in a humidified atmosphere with 5% CO_2_ to allow cell adhesion to the well. Once attached, the culture medium was removed and sample solutions were added at concentrations of 512, 256, 128, 64, 32, 16, 8, and 4 *μ*g mL^−1^, starting from the stock solution of DMEM. The final volume in each well was 100 *μ*L and the concentration of cells present in each well was 1 × 10^4^ cells. The final concentration of methanol in each well did not exceed 3%. The controls of cell growth, solvent (methanol), samples and negative control (100% lysed cells), were also prepared. The plates were incubated for 48 h at 37°C in a humidified atmosphere with 5% CO_2_. Next, 10 *μ*L of developing solution (3 mM resazurin in PBS) was added to each well [29] and the plate was incubated again for 24 h under the same conditions. Readings of absorbance at 594 nm were performed in a microplate spectrophotometer (Camberra-Packard). Assays were performed in triplicate and the absorbance values for each concentration were used to calculate cell viability according to the growth control. The CC_50_ (concentration at which 50% of the cells are viable) was calculated from the non-linear regression graph generated by plotting percentage of the cytotoxicity versus the tested concentrations [30].

A relationship between cytotoxicity and antimicrobial activity was determined through the selectivity index (SI), which was calculated by the logarithm of the ratio of the cytotoxic concentration (CC_50_) and the MIC value for microorganisms (SI = log⁡[CC_50_]/[MIC]). Positive value represents more selectivity against microorganisms than Vero cells and negative values indicate higher toxicity to Vero cells and low selectivity to the bacteria [31].

### 2.7. Statistical Analysis

Experimental results of essential oils yields were expressed as Mean ± SD for analysis performed in triplicate. Statistical analysis of the data was performed by Student's* t*-test. Analysis of Variance (ANOVA) followed by Tukey test was used for analysis of cytotoxic activity using SigmaPlot 11.0 software. Probability values of *P* ≤ 0.05 were considered to be significant.

## 3. Results and Discussion

### 3.1. Yield and Chemical Composition of the Essential Oils

The yields of the essential oils from leaves, inner bark, outer bark, and wood were 0.50 ± 0.01‰ (*w*/*w*), 0.20 ± 0.01‰ (*w*/*w*), 0.10 ± 0.01‰ (*w*/*w*), and 0.10 ± 0.01‰ (*w*/*w*), respectively. Relative amounts (% peak area) of each one of the 92 identified compounds are presented in Table 1, according to their elution order. They comprised a range of 85%–95% of total oil contents. Table 1 also shows the different chemical class of the volatile components found in the different essential oils. As expected, the overall volatile composition of leaves, inner and outer bark, and wood EOs of* K. coriacea* showed both qualitative and quantitative differences. Anyway, the leaves and inner bark EOs profiles were similarly characterized by the predominance of sesquiterpenes hydrocarbons, while outer bark and wood EOs were similarly characterized by long chain alkanes and fatty acids derivatives. In respect to the oxygenated sesquiterpenes, this class of compounds is present in high quantities in the leaves, inner and outer bark EOs, and absent in the wood EO.

Up to now, only one phytochemical study on the essential oil composition of* K. rugosa* Choisy, another specie of* Kielmeyera*, has been published [32]. In the leaves EO approximately 88% of the volatile constituents were sesquiterpene hydrocarbons, which is the same content for* K. rugosa* Choisi. The main compounds were germacrene-D (24.2%), (*E*)-caryophyllene (15.5%), bicyclogermacrene (11.6%), delta-cadinene (4.8%), alpha-cadinol (4.5%) and tau-muurolol (4.3%). All these compounds were also found in the leaves EO of* K. rugosa* Choisi, but in low concentrations. (*E*)-caryophyllene isolated from* Cordia verbenacea's* essential oil showed good results when used in the treatment of buccal organisms [3] and inflammatory diseases [33]. The concentrations here found to the compounds (*E*)-caryophyllene and germacrene-D are in the same range to those found in the essential oil of leaves and flowers from other species collected in the Atlantic Rainforest from Minas Gerais State, Brazil [34]. Thus,* K. coriacea* from the cerrado biome constitutes also an alternative source for these compounds. The compounds* alpha*-cubebene,* alpha*-copaene, and (*E*)-caryophyllene occur in leaves of* K. coriacea* and in the essential oils of leaves and flowers of* K. rugosa* Choisy [32]. However, bicyclogermacrene was not found in the leaves of* K. rugosa*. Fatty acids and long-chain alkanes were absent in the leaves EO of* K. coriacea*, but not in* K. rugosa*.

In the essential oil of the inner bark, the sesquiterpenes hydrocarbons were the main constituents, representing about 75% of the essential oil, all of them with recognized antimicrobial properties [3]. The major component was* alpha*-copaene (14.9%). Other components identified in high concentrations were* alpha*-(*E*)-bergamotene (13.0%),* beta*-bisabolene (9.5%), (*E*)-*beta*-farnesene (8.6%),* alpha*-curcumene (7.4%), and* beta*-cedrene (5.2%). Three isomers of bisabolol (*beta*-bisabolol,* epi*-*alpha*-bisabolol, and* alpha*-bisabolol) were found, making up about 5% of the essential oil. Bisabolol is a nontoxic substance that has anti-inflammatory, antibacterial, and healing properties. Therefore, it is mainly used in pharmaceuticals industry. Because of its anti-inflammatory properties, it reduces the redness of the skin caused by excessive exposure to ultraviolet (UV) rays of the sun. Through the antibacterial action, bisabolol is also used to treat bromhydrosis, decreasing the concentration of bacteria in the affected areas [35].

The two major volatile components of the outer bark oil were unidentified oxygenated sesquiterpenes (compounds 54 and 55 in Table 1), together making up approximately 13% of the essential oil. Alkanes were the compounds present at the highest concentrations (29.9%). This result is greater than that found for the bark essential oil of* Inga laurina* (Sw.) Willd collected in the same dry season [11]. Some of them were identified as hexacosane (3.0%), heptacosane (3.7%), octacosane (5.0%), and nonacosane (5.8%).* Alpha*-eudesmol (4.2%) and* beta*-oplopenone (3.8%) were the identified oxygenated sesquiterpenes with the highest concentration in this oil.

The essential oil of the wood constituted mainly of alkanes and fatty acids. Palmitic acid (16.2%) was the major component, and the main alkanes identified were nonacosane (9.7%), octacosane (8.4%), heptacosane (7.4%), and hexacosane (4.8%). Industry uses palmitic acid to produce a great diversity of everyday goods, such as soap and food additives. The compounds 2-ethylhexyl-3-(4-methoxyphenyl)-2-propenoate (*Z* and* E* isomers, 2.2%, and 2.3%, resp.) were identified in this study in higher concentrations than those observed in oils from Rutaceae species [36]. These compounds, known as octyl methoxycinnamate, are slightly viscous liquids and they are colourless and odourless. They present pharmacologic activity due to strong UVB absorbance and, therefore, are used in many formulations of sunscreens [37, 38]. Thus,* K. coriacea* from the cerrado biome constitutes also an alternative source of important bioactive compounds.

### 3.2. Antimicrobial and Cytotoxic Activities of Essential Oils

In order to observe biological activity for the EOs of* K. coriacea* the antimicrobial activities (MIC) of leaves, inner bark, outer bark, and wood EOs and their cytotoxicity against some aerobic/anaerobic oral microorganisms were tested (Table 2).

From Table 2, only the anaerobic bacteria* P. nigrescens* was significantly inhibited by all essential oils studied, with MIC values ranging from 200 to 50 *μ*g mL^−1^. According to Holetz et al. [39], MIC values less than 100 *μ*g mL^−1^, the antimicrobial activity can be considered good; from 500 to 100 *μ*g mL^−1^ moderate; from 1000 to 500 *μ*g mL^−1^ weak; and over 1000 *μ*g mL^−1^ inactive. Thus, the inner bark essential oil was the most effective against anaerobic bacteria* P. nigrescens* (MIC = 50 *μ*g mL^−1^). This result can be explained by the presence of secondary metabolites, as sesquiterpenes present at high concentrations (74.9%), including* alpha*-copaene (14.9%),* alpha*-(*E*)-bergamotene (13.0%) and* beta*-bisabolene (9.5%) which have antimicrobial activity against Gram-positive and Gram-negative human pathogens [4]. The other EOs also exhibited promising antimicrobial activity to* P. nigrescens* and, in general, weak antimicrobial activity to the others anaerobic microorganisms.

Considering the antimicrobial assays against aerobic bacteria, all the EOs exhibited from good to moderated antimicrobial activity, exception to the leaves EO (weak). This last result may be related to their low concentration of oxygenated mono- and diterpenes.

The outer bark EO (rich in long-chain alkanes) and wood EOs (rich in fatty acids and also in long-chain alkanes) presented same results with regards to anaerobic microorganisms, but not to aerobic. The literature does not present studies about the biological activities of long-chain alkanes separately. Probably, these two classes of compounds with different polarity characteristic act in synergistic effects [1]. According to Crockett [40] plants of the family of Clusiaceae (Guttiferae) have EOs rich in components hydrophobic in nature, which may explain the results of the barks EOs. The two oxygenated sesquiterpene present in high concentrations in these two essential oils were not identified (compounds 54 and 55, Table 1); however, these kinds of secondary metabolites, which correspond to 32.9% of the oil, have antimicrobial activities [1, 2, 4]. In general, the antimicrobial activity presented by the inner and outer bark essential oils can be considered promising for the prevention of dental caries and others oral pathologies in the same way as observed to the ethanolic extract and cyclohexene fraction of* K. coriacea*.

The results observed for wood essential oil against aerobic bacteria (MICs of 100 *μ*g mL^−1^, Table 2) were lower than those found for* Cassia bakeriana*, which inhibited the growth of* S. mutans*,* S. mitis* and* S. sanguinis* with MICs of 2000, 500 and 1000 *μ*g mL^−1^, respectively [11]. Fatty acids compose 22.0% of the wood essential oil and they have antimicrobial activity [41, 42]. Palmitic acid present at the highest concentration (16.2%) in the wood EO is an active antimicrobial agent present in human skin lipid samples, which can control bacterial microbiota [42]. Additionally, EO of other species of plants rich in terpenes, long-chain alkanes, and fatty acids showed strong inhibition of growth of aerobic and anaerobic oral bacteria [1, 4].

The CC_50_ values for cytotoxicity assays of the EOs are shown in Table 2. A relationship between cytotoxicity and antimicrobial activity was established through the selectivity index (SI). The leave's EO showed a CC_50_ value of 168 *μ*g mL^−1^, and MIC values of 200 and 400 *μ*g mL^−1^. The selectivity indexes are negative (−0.076 and −0.38, resp.) and, therefore, this essential oil is considered toxic for Vero cells; that is, it is not selective for the bacteria tested. The inner bark essential oil has a CC_50_ value of 74 *μ*g mL^−1^, which is also toxic for Vero cells (the SI values were −0.13, −0.43 e, and −0.73 for concentrations of 100, 200, and 400 *μ*g mL^−1^, resp.), except for* P. nigrescens,* for which the oil presented a MIC value of 50 *μ*g mL^−1^ and a positive value (0.17) of SI.

More selective results were shown by outer bark EO. In this case, toxicity was observed only for bacteria* A. naeslundii* and* B. fragilis* (the SI value was −0.50). For MIC value of 100 *μ*g mL^−1^ the SI value was 0.10. Finally, wood EO was highly selective for aerobic bacteria and for anaerobic bacteria* P. nigrescens*. The SI values for concentrations of 100, 200, and 400 *μ*g mL^−1^ were 0.71, 0.41, and 0.11, respectively. For bacteria* A. naeslundii *and* B. fragilis*, the results do not allow any inference. Long-chain alkanes and fatty acids could be responsible for the low toxicity observed for the wood EO.

## 4. Conclusions

The chemical characterization of the volatiles of the specie* K. coriacea* fills a gap in the knowledge of* Kielmeyera* genus. The EO from leaves and inner bark is rich in sesquiterpenes hydrocarbons and oxygenated sesquiterpenes. The outer bark EO is composed mainly of oxygenated sesquiterpenes and long-chain alkanes, while wood EO is rich of long chain alkenes and fatty acids. The inner bark EO exhibited remarkable antimicrobial activity against the anaerobic microorganism* P. nigrarescens*. It could be concluded that the antimicrobial activity of essential oils from* K. coriacea* against oral pathogens is promising as natural antimicrobial agents. The positive values of IS indicate that the EOs from* K. coriacea* showed greater inhibition of bacterial growth and are more selective for microorganisms than cytotoxic to Vero cells when the microorganisms were inhibited at low concentrations.

## Figures and Tables

**Table 1:** Chemical constituents of the essential oils from *Kielmeyera coriacea* Mart. & Zucc.

*Kielmeyera coriacea* Mart. & Zucc.									
Composition %									
	Compound	Leaves	Inner bark	Outer bark	Wood	AI reference	AIc	Mode of identification	Reference
1	(*E*)-hex-2-enal	0.5	—	—	—	846	847	a, c, d	e, g
2	(*Z*)-hex-3-en-1-ol	2.1	—	—	1.3	850	851	a, c, d	e, g
3	Linalool	0.4	—	—	—	1095	1093	a, c	e
4	*Delta*-elemene	0.6	—	—	—	1335	1327	a, c	e
5	*Alpha*-cubebene	0.6	—	—	—	1345	1341	a, c, d	e, g
6	*Alpha*-longipinene	—	1.2	—	—	1350	1349	a, c	e
7	*Alpha*-copaene	3.6	14.9	2.1	—	1374	1375	a, c	e
8	*Beta*-bourbonene	1.6	—	—	—	1387	1384	a, c	e
9	*Beta*-elemene	1.3	—	—	—	1389	1387	a, c	e
10	Sesquithujene <7-*epi*>	—	1.7	—	—	1390	1389	a, c	e
11	*Beta*-cedrene	—	5.2	0.9	—	1419	1418	a, c	e
12	n.i. (*M* = 204)	—	3.6	—	—	—	1419	—	—
13	(*E*)-caryophyllene	15.5	—	—	—	1417	1422	a, c	e
14	*Cis*-thujopsene	—	4.2	0.8	—	1429	1429	a, c, d	e, g
15	*Alpha*-*E*-bergamotene	—	13.0	0.8	—	1432	1434	a, c	e
16	Aromadendrene	0.9	—	—	—	1439	1442	a, c	e
17	n.i. (*M* = 204)	—	2.4	0.7	—	—	1450	—	—
18	(*E*)-*beta*-farnesene	—	8.6	—	—	1454	1454	a, c	e
19	*Alpha*-humulene	3.0	—	—	—	1452	1456	a, c	e
20	*Beta*-acoradiene	—	1.3	—	—	1469	1464	a, c	e
21	*(Z)*-cadina-1(6),4-diene	0.5	—	—	—	1461	1465	a, c	e
22	*Beta*-chamigrene	—	1.3	1.0	—	1477	1475	a, c	e
23	*Alpha*-curcumene	—	7.4	0.7	—	1479	1479	a, b	e
24	Germacrene-D	24.2	—	—	—	1484	1483	a, c	e
25	*Beta*-selinene	0.4	—	0.5	—	1489	1490	a, c	e
26	Pentadecane	—	—	0.5	—	1500	1493	a, b	e
27	*Alpha*-muurolene	—	0.6	1.1	—	1500	1497	a, d	e, f
28	Bicyclogermacrene	11.6	—	—	—	1500	1500	a, c	e
29	*Alpha*-chamigrene	—	1.7	0.3	—	1503	1500	a, c	e
30	n.i.	—	—	2.3	—	—	1502	—	—
31	*Beta*-bisabolene	—	9.5	—	—	1505	1504	a, c	e
32	*Beta*-curcumene	—	0.5	—	—	1514	1507	a, b	e
33	*Gamma*-cadinene	1.1	—	—	—	1513	1511	a, c	e
34	(*Z*)-*gamma*-bisabolene	—	1.6	—	—	1514	1511	a, b	e
35	*Delta*-cadinene	4.8	2.2	—	—	1522	1520	a, c	e
36	Spathulenol	1.1	—	—	—	1577	1576	a, c	e
37	Caryophyllene oxide	3.3	—	—	—	1582	1581	a, c, d	e, g
38	Viridiflorol	3.1	—	—	—	1592	1592	a, c	e
39	Widdrol	—	—	0.8	—	1599	1599	a, c	e
40	*Beta*-oplopenone	—	—	3.8	—	1607	1602	a, b	e
41	Oxygenated sesquiterpene	2.2	—	—	—	—	1623	—	—
42	Oxygenated sesquiterpene. (*M* = 220)	—	—	2.4	—	—	1624	—	—
43	*Gamma* eudesmol	—	—	0.8	—	1630	1631	a, b	e
44	*Tau-*muurolol	4.3	—	—	—	1640	1639	a, b	e
45	*Alpha*-cadinol	4.5	—	—	—	1652	1651	a, c	e
46	*Alpha*-eudesmol	—	—	4.2	—	1652	1653	a, c	e
47	Dioctyl eter	—	—	—	1.2	—	1656	c, d	f
48	*Beta*-bisabolol	—	1.4	—	—	1674	1668	a, c	e
49	Cadalene	—	—	1.8	—	1675	1670	a, c, d	e, g
50	*Epi*-*alpha*-bisabolol	—	1.8	—	—	1683	1680	a, b	e
51	*Alpha*-bisabolol	—	1.9	—	—	1685	1682	a, c, d	e, f
52	Oxygenated sesquiterpene	—	—	2.7	—	—	1709	—	—
53	Oxygenated sesquiterpene	—	—	2.3	—	—	1722	—	—
54	Oxygenated sesquiterpene	—	—	6.4	—	—	1731	—	—
55	Oxygenated sesquiterpene	—	—	6.3	—	—	1740	—	—
56	Oxygenated sesquiterpene	—	—	1.6	—	—	1758	—	—
57	Cyclocolorenone	—	—	1.6	—	1759	1759	c	e
58	n.i.	—	—	2.7	—	—	1764	—	—
59	Tetradecanoic acid	—	—	—	1.6	1768	1764	c, d	f, g
60	n.i.	—	2.3	—	—	—	1789	—	—
61	Cyclohexadecane	—	—	—	2.5	1880	1874	a, c	g
62	Nonadecane	—	—	1.0	—	1900	1900	a, c, d	e, g
63	Hexadecenoic acid	—	—	—	1.2	1953	1925	d	f, g
64	Palmitic acid	—	0.7	1.0	16.2	1959	1943	c, d	f, g
65	Eicosane	—	—	1.4	—	2000	2000	a, c, d	e, g
66	*Epi*-13-manoyl oxide	—	—	1.7	—	2009	2042	a, c	e
67	Kaurene	—	—	2.3	—	2042	2063	a, d	e, g
68	Phytol + n.i.	3.2	—	—	—	2110	2078	d	d, g
69	Octadecanol	—	0.6	—	4.0	2077	2100	a, b, c	e
70	Heneicosane	—	—	1.1	—	2100	2100	a, d	e, g
71	*Gamma*-palmitolactone	—	0.7	—	—	—	2118	—	e
72	Linoleic acid	—	—	—	1.2	2132	2149	c	f, g
73	Oleic acid	—	—	—	1.8	2141	2152	c, d	f, g
74	(*Z*)-2-ethylhexyl-3-(4-methoxyphenyl)-2-propenoate (*M* = 290.4)	—	—	—	2.2	—	2171	c, d	f
75	Geranyl linalool	—	0.8	—	—	—	2192	c	f, g
76	Unsaturated hydrocarbon	—	—	—	1.4	—	2192	—	—
77	Docosane	—	—	0.9	—	2200	2200	a, c	e, f
78	Octadecyl methoxy acetate	—	—	—	1.4	—	2212	d	—
79	Tricosane	—	—	1.1	1.3	2300	2300	a, c	e
80	(*E*)-2-Ethylhexyl-3-(4-methoxyphenyl)-2-propenoate	—	—	—	2.3	—	2303	c, d	f
81	Unsaturated hydrocarbon	—	—	—	1.5	—	2306	—	—
82	Oleic acid amide	—	—	—	2.4	—	2328	c	—
83	Tetracosane	—	—	1.3	1.5	2400	2400	a, c, d	e, g
84	Pentacosane	—	—	2.7	3.2	2500	2500	a, b	e, g
85	Hexacosane	—	—	3.0	4.8	2600	2600	a	g
86	Heptacosane	—	—	3.7	7.4	2700	2700	a	g
87	Octacosane	—	—	5.0	8.4	2800	2800	a, d	g
88	Squalene	—	—	1.7	1.5	—	2851	d	f, g
89	Saturated hydrocarbon	—	—	—	1.3	—	2874	—	—
90	Nonacosane	—	—	5.8	9.7	2900	2900	a, c, d	f, g
91	Saturated hydrocarbon	—	—	2.4	7.4	—	3059	—	—
92	Saturated hydrocarbon	—	—	—	6.3	—	3315	—	—

	Total	**94.4**	**91.1**	**85.2**	**95.0**				
	Yield (%w/w ± sd)	**0.5 ± 0.1**	**0.2 ± 0.1**	**0.1 ± 0.1**	**0.1 ± 0.0**				
	Oxygenated monoterpenes	0.4	—	—	—				
	Sesquiterpene hydrocarbons	69.7	74.9	8.2	—				
	Oxygenated sesquiterpenes	18.5	5.1	32.9	—				
	Oxygenated diterpenes	1.6	0.8	1.7	—				
	Long-chain alkanes	—	—	29.9	51.3				
	Fatty acids derivatives	—	1.4	1.0	22.0				
	Others	2.6	1.6	5.8	21.7				
	n.i.	1.6	7.3	5.7	—				

AI: arithmetic index on DB-5 capillary column; AIc: arithmetic index calculated.

Mode of identification: (a) Kovat index; (b) Adams mass spectral-retention index library; (c) Wiley library of mass spectral database; (d) Nist mass spectral database.

Reference: (e) Adams [20]; (f) The Pherobase [21]; (g) NIST [22].

**Table 2:** Inhibitory effect against aerobic and anaerobic oral bacteria and cytotoxic activity (CC_50_, *μ*g mL^−1^) of essential oils from *K. coriacea*.

		Minimum inhibitory concentration (MIC) – *μ*g mL^−1^				
Microorganisms		Samples of essential oils				CHD^*^
		Leaves	Inner bark	Outer bark	Wood	
Anaerobic	*Actinomyces naeslundii* ^b^ ATCC 19039	>400	>400	400	>400	1.844
	*Bacteroides fragilis* ^a^ ATCC 25285	>400	>400	400	>400	1.844
	*Prevotella nigrescens* ^a^ ATCC 33563	200	50	100	200	1.844

Aerobic	*Streptococcus mitis* ^b^ ATCC 49456	>400	100	100	100	3.688
	*Streptococcus mutans* ^b^ ATCC 25175	>400	200	100	100	0.922
	*Streptococcus sanguinis* ^b^ ATCC 10556	>400	400	100	100	0.922
	*Streptococcus sobrinus* ^b^ ATCC 33478	>400	100	100	100	0.922

Cytotoxic activity (CC_50_, *μ*g mL^−1^)						
		Leaves	Inner bark	Outer bark		Wood

*Vero cells* ATCC CCL 81		168 ± 10	74 ± 3	127 ± 1		>512

Selectivity index for Anaerobic microorganisms		−0.38; −0.38; −0.076	−0.73; −0.73; 0.17	−0.50; −0.50; 0.10		0.11; 0.11; 0.41

Selectivity index for Aerobic microorganisms		−0.38	−0.13, −0.43; −0.73; −0.13	0.10		0.71

^a^Gram-negative bacteria; ^b^Gram-positive bacteria; ^*^CHD: chlorhexidine dihydrochloride (positive control).
